# Innovative surface nanostructures to stabilize single metal site catalysts

**DOI:** 10.1093/nsr/nwaf436

**Published:** 2025-10-16

**Authors:** Yachao Zeng, Gang Wu

**Affiliations:** School of Chemical Engineering & Technology, Key Laboratory for Green Chemical Technology of Ministry of Education, Tianjin University; Collaborative Innovation Center for Chemical Science & Engineering; China; International Joint Laboratory of Low-carbon Chemical Engineering of Ministry of Education, China; Department of Energy, Environmental, & Chemical Engineering, Washington University in St. Louis, USA

Nitrogen-coordinated and atomically dispersed Fe sites in partially graphitized carbon phases, also often referred to as Fe-N-C, are the most promising platinum group metal (PGM)-free catalysts for the challenging acidic oxygen reduction reaction (ORR). They hold promise for replacing Pt catalysts in low-temperature proton exchange membrane fuel cells (PEMFCs), which are desirable for transportation and aviation [1]. Following pioneer exploration decades ago [2,3], tremendous efforts have been devoted to the development of viable Fe-N-C catalysts, aiming to elucidate active sites at the atomic level, enhance their intrinsic catalytic properties, and improve performance and durability under real operation conditions. Although significant progress has been achieved in terms of activity improvements via optimizations of the precursor and pyrolysis strategy [4], this type of attractive catalyst still suffers from significant activity loss and degradation under long-term operation, which is not yet viable for practical applications in PEMFCs. In addition to possible carbon corrosion and electrode structure/interface degradation, simultaneously enhancing intrinsic activity and stability is currently limited by a trade-off due to the co-existence of multiple types of single Fe sites, which have been clearly identified both experimentally and theoretically [5–7]. Generally, there are two primary FeN_4_ sites in such-synthesized catalysts. One is a highly active, yet unstable, pyrrolic-nitrogen-coordinated Fe site (denoted as S1 or D1 site), which is often dominant in highly active catalysts, resulting in significant activity loss during the short initial stage of operation. In contrast, the other is a highly stable but less active pyridinic-nitrogen coordinated Fe site (S2 or D2 site). Guided by this fundamental understanding, various synthesis approaches have been explored to overcome the grand challenge of the activity–stability trade-off. For example, depositing an additional thin layer of nitrogen-doped carbon onto Fe-N-C catalysts at high temperatures is effective in constructing a highly durable single-atom Fe catalyst [8]. Notably, its activity and performance are still adequate. Additionally, instead of a conventional inert atmosphere, adding a trace of H_2_ can effectively improve initial activity and fuel cell performance [9]. However, the long-term durability at desirable high voltage conditions (>0.7 V) is not yet satisfied. Therefore, eventually overcoming the activity–stability trade-off remains a great challenge in the field.

**Figure 1.**
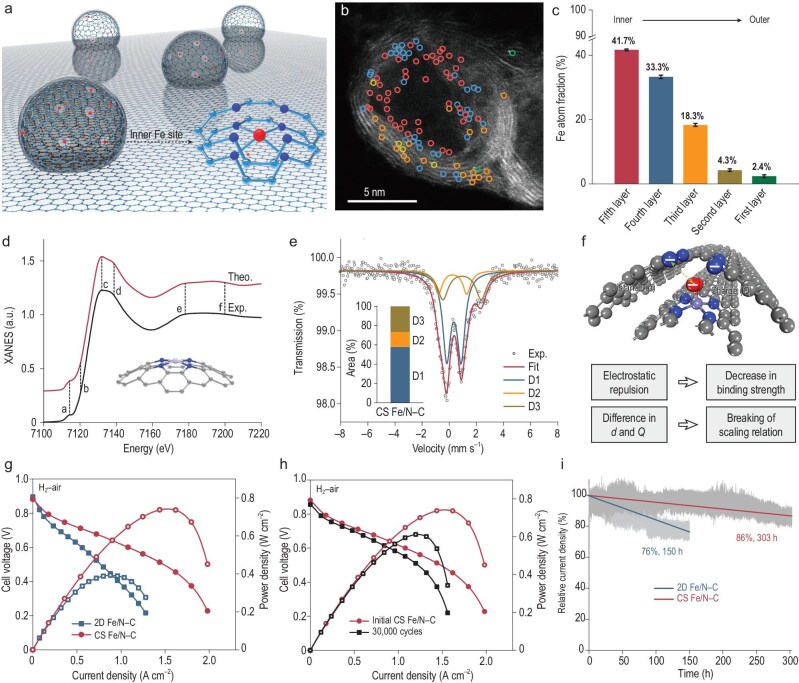
(a) Schematic illustration showing the nano-protrusion of the CS Fe/N–C catalyst. (b) High-angle annular dark field STEM image of the CS Fe/N–C catalyst with single Fe atoms (bright dots) distributed across the oriented multilayers. (c) Statistical distribution of Fe-atom fractions in the different layers of nano-protrusion. (d) Comparison between the experimental (Exp.) and theoretical (Theo.) Fe *K*-edge XANES spectrum of CS Fe/N–C; the inset indicates the structure of FeN_4_ sites in a curved surface. (e) ^57^Fe Mössbauer spectrum of CS Fe/N–C. The inset shows the ratio of D1, D2, and D3. (f) A schematic diagram showing the interaction between N atom and O atom of oxygenated intermediate in the curved surface FeN_4_ site. Grey sphere, C; blue sphere, N; mauve sphere, Fe; and red sphere, O. (g) H_2_-air fuel cell performance of 2D Fe/N–C and CS Fe/N–C under 1.0 bar H_2_-air. Specific testing conditions: cathode catalyst loading of 3 mg cm^−2^ and anode catalyst loading of 0.35 mg_Pt_ cm^−2^ for Pt/C, Nafion 211 membrane, 5-cm^2^ electrode, 80°C, 100% relative humidity, and H_2_-air at flow rates of 0.4 and 0.8 L min^−1^. (h) H_2_-air fuel cell polarization (solid symbols) and power density (hollow symbols) plots of CS Fe/N–C under 1.0 bar H_2_-air at flow rates of 0.4 and 0.8 l min^−1^ before and after 30 000 square wave AST cycles. (i) Performance retention of CS Fe/N–C and 2D Fe/N–C at a constant cell potential of 0.6 V. Reproduced from ref. [10] with permission.

Recently, in a paper published in *Nature*, Wang and his co-workers reported a durable single-atom Fe catalyst (denoted as CS Fe/N–C) with dominant S1-sites, offering an alternative approach to the durability improvement of Fe-N-C catalysts (Fig. 1) [10]. The success of the catalyst design was demonstrated via comprehensive characterizations and DFT calculations. The single-atom Fe sites are primarily hosted in nano-protrusions encapsulated by a nitrogen-doped graphitized carbon layer (Fig. 1b–e). As revealed by insightful theoretical computation, an increase in the curvature in Fe-N_4_ alongside the presence of a protective carbonaceous layer broke the linear scaling relationship in the oxygenated intermediates, due to the difference in the charge and distance between the inner and outer carbonaceous layers (Fig. 1f). The nano-protrusions contribute to the vast development of triple-phase boundaries in the novel CS Fe/N–C catalyst, achieving encouraging H_2_-air fuel cell performance and significantly enhanced durability after a standard accelerated stress test (Fig. 1g, h). In particular, the CS Fe/N–C catalyst maintained promising performance at a desirable 0.6 V over 300 hours of operation (Fig. 1i). Advanced *operando* X-ray absorption spectroscopy further revealed that the nitrogen-doped graphene layers mitigate the demetallation rates of atomic Fe sites under challenging electrochemical conditions.

In summary, the new effort to advance Fe-N-C catalysts with simultaneously improved activity and stability would provide new momentum to inspire scientists in the field to explore effective approaches to ultimately overcome the grand challenge of Fe-N-C catalysts. The continued and collective worldwide endeavor will eventually make the atomic iron catalyst viable in PEMFCs, offering sufficient performance and durability, which provides a sustainable and cost-effective route for renewable and clean energy. The innovative concepts and strategies developed in this work can be applied to other atomically dispersed single- or dual-metal site catalysts for advanced electrocatalysis, such as CO_2_ to CO conversion, with significantly improved performance and long-term durability [11].
